# Antimicrobial Use Surveillance Indicators for Finfish Aquaculture Production: A Review

**DOI:** 10.3389/fvets.2021.595152

**Published:** 2021-03-11

**Authors:** Jacob A. Narbonne, Brian R. Radke, Derek Price, Patrick C. Hanington, Amreen Babujee, Simon J. G. Otto

**Affiliations:** ^1^Human-Environment-Animal Transdisciplinary Antimicrobial Resistance Research Group, School of Public Health, University of Alberta, Edmonton, AB, Canada; ^2^Faculty of Veterinary Medicine, University of Calgary, Calgary, AB, Canada; ^3^British Columbia Ministry of Agriculture, Abbotsford, BC, Canada; ^4^Department of Fisheries and Oceans, Government of Canada, Vancouver, BC, Canada; ^5^School of Public Health, University of Alberta, Edmonton, AB, Canada; ^6^Antimicrobial Resistance (AMR) One Health Consortium, Edmonton, AB, Canada; ^7^Thematic Area Lead, Healthy Environments, Centre for Health Communities, School of Public Health, University of Alberta, Edmonton, AB, Canada

**Keywords:** antimicrobial use, antimicrobial resistance, integrated surveillance, antimicrobial stewardship, finfish aquaculture

## Abstract

Quantification and tracking of antimicrobial use (AMU) are key factors for the development of responsible antimicrobial stewardship programs and comparison between countries. Global finfish aquaculture growth and increased AMU creates the potential for exchange of antimicrobial resistance between aquatic and terrestrial environments, making AMU surveillance imperative for this industry. The objective of this review is to collate current literature on AMU surveillance indicators and their application to commercial finfish aquaculture production. A systematic search strategy was applied to five databases: Medline, Embase, Agricola, CAB abstracts, and Biosis. To be included, studies must report on at least one AMU surveillance indicator for use in animals. There is no single, standardized indicator suitable to report finfish aquaculture AMU. The type and availability of finfish aquaculture data presents unique considerations for AMU reporting. Ultimately, the indicator used should be fit-for-purpose to satisfy the objective of the surveillance program, motivation for comparison and provide useful information to the industry stakeholders. Finfish aquaculture total annual slaughter weight allows estimation of biomass for the population correction unit (PCU) to report annual total mg of active antimicrobial ingredient per PCU. These data are commonly reported by finfish aquaculture-producing countries, allowing for international comparisons. However, this precludes the ability to compare to terrestrial livestock where the PCU is based on animal numbers and an average treatment weight, which are not available for finfish aquaculture. The mg per adjusted PCU indicator provides an interesting alternative that incorporates the length of the marine grow-out phase for finfish, but is subject to the same limitations. The number of defined daily doses animal per animal-days-at-risk is useful but also limited by a lack of a defined average treatment weight. The concept of average treatment weight remains challenging for the industry as it does not accurately reflect the timing of actual AMU to fish in the system. The term “average biomass” is more reflective of the intent of AMU surveillance indicators. Defining an average treatment weight, or average biomass, will require industry engagement, which is crucial if AMU reporting is to be deemed credible and provide value back to the finfish aquaculture industry.

## Introduction

Quantification and tracking of antimicrobial use (AMU) are key factors in the development of responsible antimicrobial stewardship policy and programs (1). Antimicrobial use in livestock production has been linked to increased selective pressures for resistance to various antimicrobial drugs (AMDs) in bacteria in both agricultural and human settings (2). This spread of antimicrobial resistance (AMR) between human and animal settings can be due to environmental contamination (3, 4) or direct transmission of resistant bacteria or the genes that encode for AMR (5, 6). With the growing threat of AMR, stakeholders require robust, standardized methods to monitor and report AMU in food animal agriculture (7). The threat of trade restrictions from AMR (8) and the need to assess and promote antimicrobial stewardship means that countries and agricultural industries must be able to compare their AMU in a standardized and robust manner (9).

The worldwide growth of finfish aquaculture production has resulted in marked increases in therapeutic and prophylactic use of antimicrobial drugs in marine and freshwater settings (10–12). This presents the opportunity for exchange of resistance determinants between water and terrestrial environments (13). The potential for environmental exposure to AMR organisms and genes from finfish aquaculture operations poses a unique One Health threat (13). These resistance determinants can spread from farmed to wild fish populations and antimicrobial residues from fish feeds can settle in the benthic zone of the ocean (13, 14). As a result, surveillance of AMR that can integrate AMU data from the finfish aquaculture industry is of paramount importance to guide future stewardship efforts. Application of AMU surveillance indicators to finfish aquaculture AMU data will be pivotal to inform future stewardship programs and allow for international comparisons.

As AMR continues to be a preeminent One Health threat, indicators for analyzing and reporting AMU are increasingly important tools. The design and improvement of AMU surveillance programs must consider these indicators to best promote antimicrobial stewardship (15–17). Some European Union (EU) countries (Denmark and the Netherlands) set and enforce AMU benchmarks for livestock production that rely on AMU data and indicators (18, 19). However, most indicators have been developed for terrestrial food animals. There is no international consensus on the preferred, standardized AMU indicators for terrestrial animals, let alone finfish aquaculture species. It is widely recognized that the purpose of surveillance directly affects the choice of surveillance indicator and subsequent data collection (16). Regardless of the policy intent of collecting AMU data, which can range from informing industry-driven stewardship programs to benchmarking and between-country comparisons, robust indicators are required.

Government organizations create and define AMU indicators because they typically bear the responsibility of monitoring AMU over time (17), but the outputs of surveillance must be fit for the purposes of both government and agriculture industries. The lack of standardized indicators makes it difficult to compare AMU between different countries and species. This is further compounded by differences in items important for their derivation, such as animal average treatment weights (ATWs), defined dosing standards, and variable production practices and animal cycle lengths between countries (20, 21). In addition to this lack of standardization and comparability, all AMU indicators suffer from their own respective limitations based on poor data availability, or uncertain assumptions such as animal weights and drug label and used doses (22). Regulators must be transparent in how indicators are derived and used to clearly reflect the burden of AMU in a population and allow for comparison. A recent publication, based on a 2016 literature search, reviewed and categorized commonly used AMU indicators for food animal production (9). The objective of this review is to collate current literature on AMU surveillance indicators and to consider how these can be applied to AMU surveillance in commercial finfish aquaculture production, with specific focus on the Canadian finfish aquaculture context.

## Review Methodology and Results

A systematic search string was developed with the assistance of a librarian. The complete search strategy and results can be found in Supplementary Material. Published articles were obtained by executing the search on January 20, 2020 in five scientific databases: Medline via Ovid®, Agricola® *via* ProQuest®, CAB Abstracts *via* Web of Science™, Biosis® via Web of Science™, and Embase via Ovid®. Key search words were broken into five categories in order to capture articles of interest: surveillance/monitoring, antimicrobials, use, metrics/indicators of interest, and animal species of interest. Searches were limited to January 1st, 2016 onwards in order to capture literature not covered by the recent review (9). Articles were then sorted and screened based on defined inclusion and exclusion criteria. To be included, studies must report on at least one AMU surveillance metric or indicator for use in animals. Studies were excluded if they did not include discussion of an AMU surveillance metric or indicator, if they did not discuss AMU surveillance in animals, if they were not written in English, or if they were theses or dissertations. All articles were screened at two levels by one reviewer (JN). All articles were managed, deduplicated and screened using Mendeley® (Elsevier, 2020). First level screening included titles and abstracts. Second level screening included full article text. Government and intergovernmental/international reports on AMU metrics and indicators in livestock and finfish aquaculture settings were identified based on investigator knowledge. Supplementary articles and reports were identified by hand-searching the reference lists of included articles and knowledge of the investigators. See Supplementary Material for the complete results of database searches and article screening. Supplementary Figure 1 includes the detailed results of the search and screening. There were 1,660 articles after deduplication, of which 38 progressed to second-level screening. A total of 27 articles (20 peer-reviewed and seven governmental reports) were included in the final review. A complete list of articles with extracted data are included in Supplementary Table 2.

## An Overview of Antimicrobial Use Metrics and Indicators

The use of the term metric vs. indicator varies in the AMU surveillance literature. Some use the terms interchangeably, while others differentiate between them and define an AMU metric to be a technical unit of measurement (e.g., frequency of use) and an indicator to be an AMU measurement in relation to a denominator such as animal biomass, population size or time unit (17). This becomes confusing when considering, for example, the Population Correction Unit (PCU) and Defined Daily Dose Animal (DDDvet) for a given antimicrobial (17, 20, 23, 24). The PCU and DDDvet, by definition, are not AMU metrics because they do not quantify AMU. They are, however, useful population or drug-specific metrics that are used to derive AMU indicators. For the purpose of this review, we focus on indicators as the estimate of AMU that standardizes a measure of the frequency of use by a denominator with consideration of their application to finfish aquaculture production. All AMU indicators are reported over a period of time (typically 1 year, unless otherwise specified) and another unit or combination of units to standardize use by the population being considered. Sometimes these denominators include technical units of measurement specific to the population and/or drug in question.

Werner et al. (9) considered two overarching categories of commonly used AMU indicators based on quantity of AMD used and the course of AMD application. Quantity-based indicators characterize the amount of AMU in terms of the weight of AMD distributed, sold or administered/used per kg of body weight, standardized weight, or number of doses used. Course-based indicators specify if and how often AMU occurred by estimating the number of drug treatments or courses an animal or group of animals receives over time (9, 25). For this review, we consider the terms AMD, “drug” and “active ingredient” to mean a single active antimicrobial ingredient, to be distinguished from antimicrobial products that contain more than one active ingredient. A dose of active ingredient is the amount of AMD administered in a single application whereas dosage is the amount of AMD administered per kilogram of bodyweight (9). However, the terminology in the literature is not consistent in the use of the dose vs. dosage. A treatment is all administrations of an AMD given to one animal in 1 day (9). A course is a full regimen (the number of days) of treatment with an AMD as outlined by the instructions on the drug label (1). Table 1 includes examples of AMU and population metrics used to derive the resulting AMU indicators.

**Table 1:** Examples of antimicrobial use (AMU) surveillance indicators linked to underlying AMU and population metrics.

	**AMU or population metrics**	**AMU indicators**
Quantity-based indicators	Weight of active ingredient	
	Biomass—Population Correction Unit (PCU)	Total weight/PCU
	Biomass—Adjusted PCU (APCU)	Total weight/APCU
	Number of animals	Total weight/number of animals
	Defined daily dose animal (DDDvet)	Number of DDDA (nDDDvet)
	Used daily dose animal (UDDA)	Number of UDDA (nUDDA)
	UDDA	Treatment frequency (TF)
	DDDvet and PCU or APCU	Treatment incidence (TI)^*^
Course-based indicators	Defined course dose animal (DCDvet)	Number of DCDA (nDCDvet)
	DCDvet and PCU or APCU	Treatment incidence (TI)^*^

* *Quantity or course-based definition of Treatment Incidence depends on the metric used to derive the indicator*.

### Total Weight of Active Antimicrobial Ingredient

The total weight of active ingredient for AMDs used for a population over a given period of time (usually 1 year) is a rudimentary measure of AMU (25). It simply relies on collecting and collating the total amount of active ingredient distributed, sold or administered/used over a period of time. The Canadian Integrated Program for AMR Surveillance (CIPARS) reported total annual weight of AMDs distributed for animal use for over a decade (26). Future reporting will be broken down by province and animal species (e.g., finfish aquaculture) (27). The 2017 DANMAP (Danish Integrated AMR Monitoring and Research Program) report included the total annual amount of AMDs sold in kilograms to the Danish finfish aquaculture industry (28). In 2016, Norway reported total kilograms of AMDs in finfish aquaculture prescribed by finfish species, production stage, and total biomass (29).

Unfortunately, total weight of active ingredient is insufficient for AMU surveillance when used alone due to several problems inherent with the lack of standardization by drug dosage or population size and animal weight at risk. This measure can only be used to meaningfully compare the AMU of two essentially identical farms, regions or countries using the same AMDs (with identical doses) for identical livestock populations due to its tendency to inflate the AMU of a farm/country with a larger population of animals (30, 31). It can also result in erroneous comparisons of AMU between species of different sizes (e.g., chicken, fish, cattle, or humans) or with drugs with higher (e.g., tetracyclines) or lower (e.g., macrolides) mg/kg dosages (21). This measure is, however, commonly used as a numerator in other AMU indicators that standardize the total annual amount of active ingredient by the different denominators in other quantity and course-based indicators.

### Population Correction Unit and mg/PCU Indicator

The PCU is the theoretical estimate of the biomass or weight (kg) of animals that could be exposed to a given total weight of active AMD ingredient used in a country to standardize national AMD sales by a population at risk of AMU (32). It is a useful tool to pool all animal biomass to assess collective animal AMU, but can also be broken down to use and biomass per animal species. Biomass and weight are often used synonymously and by definition are the same thing, but this can be confusing when considering the PCU. Biomass in the PCU context is a population weight over a period of time (a year) (33) whereas weight implies a specific weight of an animal(s) at a specific point in time. We propose that biomass is a better representation of a population at risk of AMU over time for PCU estimation. The PCU for each species and sub-category of animal is calculated by multiplying the total number of living and slaughtered animals by a standardized theoretical weight, referred to as the average treatment weight (ATW). The European Surveillance of Veterinary Antimicrobial Consumption (ESVAC) program of the European Medicines Agency (EMA) derived the formula for the PCU shown in Equation 1 (32). The number of animals incorporates the different components of a typical production chain (Equation 1.1).

(1) $$\begin{array}{l} PCU_{species}\left(kg\;biomass\right)=number\;of\;animals\;x\;ATW\;\left(kg\right) \end{array}$$

(1.1) $$\begin{array}{l} Number\;of\;animals=animals\;present+slaughtered+\\ \;imported-exported \end{array}$$

The mg/PCU indicator (Equation 2) simply divides the total mg of AMD by the PCU estimate for the given population. This allows for the comparison of AMU between farms/countries with differing amounts of exposed animal biomass while controlling for animal demographics (34). The mg/PCU indicator standardizes the total weight of AMD distributed/sold/administered by the biomass of the animal population. When the demographics of the animal populations differ between countries, it is possible that this will influence the resulting estimates of national AMU, but the mg/PCU indicator at least accounts for variations in animal numbers and weights across populations (25). For example, it has a marked effect on the comparison of total AMU between animals and humans when one considers total weight of active ingredient comparisons vs. those using mg/PCU. Canadian AMU reporting is included in reports from both CIPARS and the Canadian AMR Surveillance System (CARSS) (35, 36). Total active ingredient reporting shows that ~80% of antimicrobials sold/distributed are for use in animals and crops, compared to 20% in humans (35–37). However, when you piece together the mg/PCU analyses for 2018, animal AMU is 1.4 times that of human use when standardizing by the respective population PCUs. These results are not explicitly included in any of the reports but were presented in CIPARS stakeholder meetings (results taken from presentations). This highlights the importance of context when considering the underlying population at risk of exposure. First, the population of animals in Canada greatly exceeds that of humans (35). This, combined with the relative sizes of, for example, cattle, chickens and humans, will impact the population PCU and the subsequent mg/PCU results when comparing humans and animals using this indicator. It is a stark contrast to the annual total active weight of active ingredient results.

(2) $$\begin{array}{l} mg/PCU_{species}\left(mg/kg\;biomass\right)=\frac{total\;AMD\;used\;\left(mg\right)}{PCU_{species}} \end{array}$$

The ATW in Equation 1 has variably been termed average treatment weight, average or estimated weight at treatment, and theoretical weight at the time most likely for treatment (32, 38). The ESVAC reports the weights they use and typically reference the original publications for these values (32, 39–42). These include body weights for categories of livestock, some of which are analogous to the animal categories used in the PCU calculations. Montforts (39) defined the term “average body weight” for animals that are reared from a starting weight onwards, compared to animals kept at their mature body weight, for which maximum body weight is used (39). Montforts and Tarazona Lafarga further proposed that body weights at treatment should be based on adult weights for mature animals and the mean of starting and slaughter weights for production animals (41). Other definitions for ATW state that these weights represent the most likely size of an animal treated with an AMD (26, 32, 34).

The interpretation of the ATW in the PCU is a common point of confusion when working with specific industry stakeholders because it does not necessarily indicate the actual treatment practice for a given food animal species or category. Different diseases and drugs are used at different points in the production cycles for different species, meaning that it truly is a theoretical “average” weight of the overall animals at risk of AMU in that population for a given period of time rather than the typical weight at treatment. For example, most farmed Atlantic salmon in western Canada with yellow mouth, caused by *Tenacibaculum maritimum*, are treated with antimicrobials early in the marine grow-out phase when they are well below their mean marine production weight (43). Use of the constant body weight of mature livestock as the ATW is eminently reasonable. However, for animals reared for slaughter there is a lack of evidence or rationale to support that antimicrobials are typically administered at their ATW (i.e., their average weight in the population). For animals at slaughter, the term ATW can be confusing in the interpretation of the PCU calculation. Average biomass is more synonymous with the average weight of an animal in the population that is at risk of AMU. It is a more accurate label in the PCU calculations, rather than the term ATW. The ATW is also used to derive other indicators with the same concerns (see nDDDvet and nDCDvet below).

It is still debatable if the ATW for a given animal species (e.g., beef cattle or farmed salmon) should be constant between the populations being compared in different countries. The specified ATW can vary by country, animal species or breed (34, 44). As a hypothetical example, consider one country that produces predominantly Atlantic salmon with an average slaughter weight of 5 kg (45, 46) and an estimated ATW of 2.5 kg (41). The comparator country produces mostly Pacific Coho salmon, with an average slaughter weight of 2.5–3.5 kg and ATW of 1.25–1.75 kg (47). Using a constant ATW of 2.5 kg allows for an apparent “apples-to-apples” comparison of AMU between these countries. However, the numbers may not accurately reflect the population demographics of the second country. This is evident in the CIPARS reporting of AMU that compares mg/PCU estimates for terrestrial livestock using either ESVAC or Canadian-derived ATWs (26). Supplementary Table 1 shows a comparison of ATWs used by ESVAC and those averaged over 28 European countries (34). If animal reporting is available by weight group or production type within a given species, then a more accurate estimate of ATW and PCU is possible. CIPARS reports two mg/PCU indicators derived using the ESVAC defined ATWs and the Canadian ATWs agreed upon by animal commodity groups (35). This allows for comparison between the EU and Canada using a common ATW and a more Canadian representation of AMU based on Canadian ATWs.

For terrestrial species, ESVAC uses the reported numbers of existing and slaughtered livestock from European countries for Equation 1.1, and CIPARS uses Canadian numbers (26, 32). However, finfish production is typically reported by total annual slaughter weight and does not include reporting of animal numbers by any country or producing entity in the world (32). Some regions, such as Chile and the Canadian province of British Columbia, report total annual slaughter weights by Atlantic and Pacific salmon species (48, 49), while others (Canada overall, Norway, and the UK) report total finfish aquaculture slaughter weight without species breakdown (50–52). International reports of finfish aquaculture are also available from the Food and Agricultural Organization (FAO) Fisheries and Aquaculture Department (53) and its subsequent FishStatJ app (54), both of which rely in part on industry reporting from producing nations/regions. As ESVAC does not specify an ATW for farmed finfish or the number of fish slaughtered, they report the farmed finfish PCU as the total annual slaughter weights (55). This is consistent with 2018 OIE determination of PCUs for finfish aquaculture (56). The rationale for finfish PCU departing from the terrestrial animal PCU approach of using animal numbers and ATW is unclear, other than data availability, as is the effect of this departure on the resulting national PCUs and mg/PCU indicators for AMU. A recent study by Schar et al. (57) reported current and projected trends in global aquaculture AMU using the mg/PCU metric where the PCU was also based on total annual production weights by species class (57) from the FAO FishStat data (54).

As an example, Atlantic salmon make up about 96% of recent finfish aquaculture production in British Columbia, with an average slaughter weight of 5 kg (58). A reasonable estimate of ATW is approximately half of this (2.5 kg), based on the method Montforts and Tarazona Lafarga (41) using the mean of initial (0 kg) and final weight (5 kg). The number of fish can be estimated by dividing the total annual slaughter weight by the 5 kg average slaughter weight. The number of fish and the average animal weight of 2.5 kg can then be used to derive the PCU. The net effect of these calculations is that the finfish PCU biomass (which is analogous to the PCU biomass of terrestrial species) is half the total annual finfish slaughter weight. Either method assumes that only slaughtered fish are eligible for AMD treatment and ignores the live fish (brood-stock and early growing fish) and fish mortality, which can range from 5 to 15% (59). This is similar to poultry where the PCU includes only slaughtered broilers and turkeys, but does not include breeder flocks (26). In British Columbia, the average grow-out length of Atlantic salmon can range from 18 to 24 months (45, 46, 60), meaning that the annual slaughter weight also does not account for the animals in early growing phase of production. It also relies on the standard slaughter weight for the fish in question, which could vary by fish species and production region or country. Grow-out cycle length, and sometimes slaughter weight, is highly dependent on degree days from water temperature and varies by oceanic region (61). The marine grow-out cycle occurs in saltwater and ignores the freshwater phase of production for salmon and finfish where this applies, a feature unique to the finfish aquaculture industry. However, if applied in the same manner to different populations for comparison, the PCU_finfish_ metric is useful for relative comparison of AMU between countries. Like CIPARS, one can consider using region and/or species-specific slaughter weights and ATWs to show the relative comparisons of using the same values vs. regional specific values (26).

The mg/PCU indicator is adept at identifying low and high users of AMDs due to its straightforward interpretation when the comparison groups have similar animal species demographics (30). However, the comparison of AMU between farms/countries can be problematic due to either under or over-representing AMU across operations with differing ATW (e.g., cattle in North America vs. Europe) (21, 25). These can vary between countries, regions and farms as the result of different breeds and variable production practices such as feeding protocols (1). For example, while ESVAC estimates the mean slaughter cattle ATW to be 425 kg (32), the standard slaughter weight for beef cattle in Canada ranges between 500 and 640 kg (21) and averages 627 kg in 28 EU countries (34). Countries where production practices result in markedly different treated and mature animal weights should consider developing their own animal weight standards in order to make more accurate estimates of representative AMU (25), but there remains debate about how to compare these estimates between countries using different weights. This also creates the ability for a reporting country to manipulate their PCU and subsequent mg/PCU results based on the used ATWs. The indicator incorporates country-specific ATW, reducing bias from variable production practices, but must be accompanied with transparency in their specification of weights and units for weight-based metrics like mg/PCU (21, 34).

There is some concern about comparing mg/PCU estimates between countries that include all antimicrobials and all animals when there is variation between food animal production (e.g., relative levels of chicken, cattle, pigs and finfish) (34). Use of the mg/PCU indicator to compare AMU of countries using total PCU values including different livestock demographics should be interpreted with these differences in mind, as AMU intensity and duration can differ between species for reasons such as variations in production practices of the nation, or the varying length of life of different livestock species. The mg/PCU indicator treats AMU in different species or risk categories equally when this may not be the case. For example, AMU in broiler chicken production may pose a different risk of selection for and transmission of AMR through the food chain than beef cattle due to differing lifespans and timing of AMU in the production cycle (34). Another example is beef vs. dairy cattle that use different types and overall weights of AMDs in feed or by parenteral or intramammary administration at different points in the production cycle, with markedly different mg/kg drug dosages. However, the standardization using PCU biomass is more appropriate for comparison than simple total weight of active ingredient in this regard. If one desires more specific species comparisons, then mg/PCU broken down by animal species can provide this relative comparison at a specific industry level (if data are available). National surveillance from CIPARS and ESVAC reports total mg/PCU for all livestock species, but species-specific mg/PCU is possible (32, 51).

The mg/PCU indicator cannot account for AMDs with different dosages (e.g., the total mg of active ingredient will be less for a drug with a lower mg/kg dose) (21), but drug-specific mg/PCU estimates are also possible (51). Given that drug doses can vary between categories of importance to human medicine (62), use of drugs with higher doses (e.g., tetracyclines) may inflate mg/PCU estimates compared to those with lower doses (e.g., macrolides) (21). This creates difficulty in assessing the relative prudence of AMU between countries using mg/PCU that includes all AMDs and all livestock (25, 63).

The mg/PCU indicator can also be calculated using more specific animal weights, such as the actual weight of animals at the time of drug administration or actual slaughter weights of animals linked to their individual AMU, if data are available, but these are extremely difficult to procure for large populations. The census of AMU data in 2.6 million feedlot cattle in Western Canada were accompanied by actual market weight data (31), but the availability of such granular data are not commonplace for livestock surveillance systems. This method offers the advantage of accuracy for a specific population, but unless comparators are also using real data, it offers no advantage over using ATW other than an assumed increase in the real-world accuracy of the PCU denominator. A potential alternative to mg/PCU uses actual production data as a denominator instead of standard or true animal weights, such as mg AMD/100,000 head of feedlot cattle (21), mg AMD/1,000 L of milk produced in dairy operations (25), or mg AMD/10,000 broiler chickens or eggs produced in poultry operations. This removes the need for estimating ATWs of different populations of animals. However, it only allows for within-species comparisons and has also created consumer confusion that AMDs are actually present in animal products (25). The use of production data as a denominator is highly limited to the type of operation for its application as many livestock systems do not produce an easily measured quantity such as number of head, kg of milk or number of eggs.

For finfish aquaculture, producing countries (Norway, Chile, the United Kingdom, and the European Union) and the OIE report AMU in mg/PCU using total annual slaughter weight for their PCU estimate (49–51, 55, 56). Total annual slaughter weight represents the most accurate data available. It can be used to estimate the ATW as described by Montforts and Tarazona Lafarga (41) and approximate the mg/PCU method that relies on real production data that is arguably more representative of the true population of slaughtered fish (31). The total annual slaughter weight of fish is a corollary to the example of using actual production data for a denominator that is applicable for finfish aquaculture production. Using a total annual slaughter weight PCU to compare finfish AMU between countries is advantageous because there is no need to define an ATW, especially if the ATW is variable between nations. The limitations of using slaughter weight are real, but it provides the benefits of transparency and consistency. Accounting for missing fish in the population and/or estimating an ATW would simply apply a scale factor to the mg/PCU result. Consistency for the PCU estimation is key to allow for international comparison.

### Adjusted Population Correction Unit and mg/APCU Indicator

Radke proposed the adjusted PCU (APCU) as an alternative interpretation to the PCU (34). The PCU does not consider the lifespan of animals in its estimate of the biomass at risk of treatment (21, 34). Risk of animal exposure to AMU is related to their weight and length of life (34). The APCU accounts for the total weight of animals in a population and their length of life to calculate the total animal biomass for possible AMD exposure, resulting in life-adjusted weights for animal categories (Equations 3 and 3.1) (34). The consideration of an animal's average lifespan improves comparability between different species where length-of-life differs greatly, such as in the case of cattle, swine, poultry and finfish. It also accounts for the increased possibility of exposure to AMD for animals as they live longer (64).

(3) $$\begin{array}{l} APCU_{species}\left(kg\;biomass\right)=number\;of\;animals\;x\\ \;life\text{-}adjusted\;treatment\;weight\;\left(kg\right) \end{array}$$

(3.1) $$\begin{array}{l} Life\text{-}adjusted\;treatment\;weight\;\left(kg\right)=ATW\;x\;length\;of\;life\;\\ \;\left(years\right) \end{array}$$

(4) $$\begin{array}{l} mg/APCU_{species}\left(mg/kg\;adjusted\;biomass\right)=\frac{total\;AMD\;used\;\left(mg\right)}{APCU_{species}} \end{array}$$

Radke (34) found differences between PCU and APCU estimates of Canada and eight European countries when using the same number of animals for both. The APCUs for longer-lived animals (cattle) increased compared to shorter-lived animals (pigs, poultry, sheep, and goats). This has the effect of reducing the relative AMU in a long-lived species by increasing the size of the denominator in the mg/APCU indicator (Equation 4), with the opposite effect in shorter-lived species. The data required to determine length of life used by Radke (34) was obtained from the literature.

Application of the APCU to finfish is an interesting premise as the animals are small relative to cattle, but have a relatively long lifespan. However, its derivation is more difficult due to the lack of reporting of animal numbers or a defined ATW and the length of life for different finfish species. It is also still limited in that it does not account for the variable relative mg/kg doses of different AMDs. An interesting consideration is the application of the length of grow-out cycle to a total slaughter weight for finfish and derived fish numbers and biomass. Using the concept of life-adjusted weights (34), one could estimate the APCU for fish to align with the approach used for terrestrial animals. At this time, we are not aware of any governments that are using mg/APCU to report finfish AMU data. Its utility for application to finfish aquaculture requires further research.

### Defined Daily Dose Animal and Number of DDDvet Indicator

The DDDvet (also known as the DDDA), an adaptation of the human DDD, is the “assumed average dose (of antimicrobial) per kg of animal per day” of treatment (Equation 5), expressed in mg/kg/day (23, 26, 65, 66). The DDDvet was described by ESVAC and the EMA as part of an effort to develop a system for the collection of harmonized data on AMU in the European Union (23, 65, 66). By definition, the DDDvet for each active drug ingredient is specific to the EU as it represents the average dosage (the arithmetic mean) of the daily dosages of that ingredient based on the dosage labels from European countries (23, 65). The DDDvet for specific AMD active ingredients are only assigned by ESVAC if they possess an Anatomical Therapeutic Chemical classification code (67).

(5) $$\begin{array}{l} DDDvet_{drug}\left(mg/kg/day\right)=\frac{mg/kg\;drug\;dose}{day\left(s\right)\;duration\;of\;effect} \end{array}$$

(6) $$\begin{array}{l} nDDDvet_{drug}=\frac{total\;AMD\;used\left(mg\right)}{DDDvet_{drug}\left(mg/kg/day\right)\;x\;ATW\left(kg\right)} \end{array}$$

The nDDDvet indicator adjusts the weight of active ingredient distributed/sold/administered by the DDDvet of the AMD and the ATW of the animal in question (Equation 6) (20). This calculation is AMD and animal specific, making its derivation comparable between populations using similar AMDs with similar livestock demographics (44). The DDDvet for combination products including more than one active antimicrobial ingredient measure use by counting the DDDvet of each single ingredient product separately, with specific methods for synergistic combinations (9, 23, 65). The nDDDvet also accounts for long-acting parenteral formulations by dividing the single administered dose by the number of days of the duration of therapeutic effect (65). The duration of effect is an important consideration for quantifying AMU in terms of evaluating selective pressure and the development of AMR (21). To compare AMU for a population with a mixed animal demographic, species-specific nDDDvets can be calculated and summed to consider total AMU. However, comparison of total nDDDvets for all animals between countries with dissimilar populations without accounting for numbers, types and ATWs of animals is questionable for similar reasons as outlined for the mg/PCU.

The use of European standard doses is advantageous to compare AMU in European countries that operate under the customs and production practices where ESVAC standard doses apply. Current, defined DDDvets from ESVAC for AMDs exist for swine, cattle, and broiler chickens (66). While the nDDDvet offers a standardized dose-based indicator compared to the mg/PCU biomass-based indicator, it is still difficult to interpret and compare between countries with different doses, production practices, animal populations and ATWs (1). Countries outside the EU may have different approved drug label dosages that lead to country-specific DDDvet definitions. Canada has defined Canadian industry-specific DDDvetCAs for swine, chickens, turkeys and cattle (20, 24, 30). Typical drug labels for in-feed or water administration for these species use inclusion rates of mg AMD per 1,000 kg of feed or 1,000 L of water. ESVAC and CIPARS use a conversion factor to estimate the daily dose per kg animal for AMDs administered in feed or water by estimating feed or water intake (26, 65). Conversely, finfish aquaculture drug dosage labels provide a mg/kg/day values, negating the need to apply this conversion factor for standard feed intake of fish (68).

The DDDvet is a technical unit of measure that represents a compromise between all European label dosages for an AMD (23, 25, 26, 65). As a result, it is a compromise of existing dosages and is intended for reporting AMU data, but it does not necessarily reflect the daily dosages recommended or prescribed for use in animals. The nDDDvet by itself does not provide any information as to the number of animals treated or the population at risk of treatment that is provided by mg/PCU (9). It also does not provide the length of treatment (the number of days of consecutive or total therapy), the actual daily dose applied, or the total amount of AMD used. The nDDDvet also relies on defining the ATW and is subject to the same concerns that this poses for PCU estimates. Inconsistent ATWs between countries create concerns for international comparisons. This is compounded by inconsistent DDDvets between countries.

The nDDDvet can be considered for finfish aquaculture AMU, but there are important limitations. Approved label dosages exist within countries for commonly used AMDs, such as oxytetracycline and florfenicol, but neither ESVAC nor Canada have defined DDDvets for these drugs in finfish aquaculture species at this time (23, 26, 65, 66). The lack of an ESVAC or Canadian ATW for finfish also raises uncertainty for the application of nDDDvet. There are methods to estimate the ATW using the total annual slaughter weight, as described for the mg/PCU. However, there is no simple way to do this knowing that average slaughter weights and industry salmon species demographics vary between countries and regions. There is no way to incorporate total annual slaughter weight directly into the nDDDvet calculation like there is for mg/PCU. This makes the nDDDvet less transparent and subject to variation depending on the assumptions for the ATW as well as non-standard DDDvets.

### Used Daily Dose Animal and Number of UDDA Indicator

The UDDA is the daily dose of an active ingredient per animal that is typically administered to an animal (mg drug/animal/day—Equation 7) (9, 69–71). Alternatively, the UDDA_kg_ (UDDA per kg—Equation 8) is the actual administered dosage of an active ingredient per day per kg of body weight of a treated animal (9). These require the actual number of treated animals, their weights and the number of days of treatment to be known. As a result, they are based on real data rather than the theoretical value presented by the consensus of several doses that make up the DDDvet. The UDDA allows for comparisons of AMU between populations using the same active ingredient.

(7) $$\begin{array}{l} UDDA_{drug}\left(mg/animal/day\right)=\frac{total\;AMD\;used\;\left(mg\right)}{\#\;treated\;animals\;x\;\#\;treatment\;days} \end{array}$$

(8) $$\begin{array}{l} UDDA_{kg\;drug}\left(mg/kg/day\right)\;=\\ \frac{total\;AMD\;used\;\left(mg\right)}{\#\;treated\;animals\;x\;treatment\;weight\;\left(kg\right)\;x\;\#\;treatment\;days} \end{array}$$

The number of UDDA (nUDDA) indicator is the sum of daily applications in a population (Equation 9) (9). It represents the amount of actual administered AMD doses for a given animal population (9, 44). It does not indicate how much active ingredient is being used, it simply reflects the frequency of treatments with AMD (72). It requires granular data such as the number of animals treated, the number of days treatment occurred, and the number of active ingredients administered (72, 73). It is also only specific for similar populations being analyzed at a point in time using the same active ingredients for treatment (9, 20).

(9) $$\begin{array}{l} nUDDA=\#\;treated\;animals\;x\;\#\;active\;ingredients\;x\;\\ \;\#\;treatment\;days \end{array}$$

With the increased level of specificity at the farm level, indicators like nUDDA can be used as tools to show actual AMU for benchmarking between similar farms. One study compared the nDDDvet and nUDDA to quantify AMU at the farm level using similar data sets (71). There were differences in observed AMU due to the use of actual vs. standard animal weights. Comparisons between populations are further limited by a lack of standardization of the UDDA between farms, specific treated animals within populations, veterinarians, and producers (71, 73). Due to the presumed real-world accuracy of the UDDA for comparing AMU between similar populations with similar dosing practices, the ratio between the UDDA and DDDvet of AMU in a population can reflect the suitability of dosing (44). The higher the ratio between these two metrics, the more excessive the AMU is when compared to baseline expectations of AMU built into the DDDvet metric (in the form of expected average dosages). In reality, the nUDDA relies heavily on granular data for each food animal industry, making its use for comparison limited unless these data are collected at the national level. The lack of these specific data for number of treated fish and actual treatment weights in finfish aquaculture make the nUDDA a poor candidate for estimating AMU in this industry.

### Defined Course Dose Animal and Number of DCDvet Indicator

The Defined Course Dose Animal (DCDvet) does not have a human counterpart and is defined as the “average dose per kilogram of animal per species per treatment course,” or the product of the treatment length and the DDDvet for that drug (Equation 10) (1, 23, 25, 65, 66). The number of DCDvet (nDCDvet) adjusts the total weight of active ingredient by the DCDvet and ATW (Equation 11).

(10) $$\begin{array}{l} DCDvet_{drug}\left(mg/kg_{course}\right)=DDDvet_{drug}\;\left(mg/kg/day\right)\;x\;\\ \;treatment\;length\;\left(days\right) \end{array}$$

(11) $$\begin{array}{l} nDCDvet_{drug}=\frac{total\;AMD\;used\;\left(mg\right)}{DCDvet_{drug}\left(mg/kg_{course}\right)\;x\;ATW\;\left(kg\right)} \end{array}$$

Course-based indicators can give estimates on the likelihood and propensity an animal will be treated with AMDs in a specified period of time (74). They require increasingly granular data ranging from simply applying an animal-time-at-risk denominator of an overall population to finding the exact dose and number of days that animals are exposed to that dose for benchmarking a population (74, 75). These can be powerful tools in developing a broader view of AMU when used in conjunction with quantity-based indicators (30). Similar to the DDDvet, the recommended treatment/course length for the DCDvet can vary substantially between countries and on a case by case basis, depending on the animal species, diagnosis, prescriber and end-user. This influences the comparability of AMU based on nDCDvet between different populations using different treatment courses (1). Countries with proprietary and/or drug label treatment practices that differ substantially from those outlined by the ESVAC DCDvet are difficult to quantify without defined treatment courses. Antimicrobial use would be underestimated if a recommended course is shorter than its comparator group (1).

Unfortunately, unlike mg/PCU calculations, the DCDvet does not account for topical AMU applications like foot bathing and intramammary infusions (65). To this end, one UK study defined intramammary therapy as four tubes per cow to be a single course of AMD administration, but foot bathing antimicrobials were not included in nDDDvet or nDCDvet analyses (63). In another UK study, the nDCDvet was used in conjunction with nDDDvet and mg/PCU estimates of AMU on British dairy farms to incorporate intramammary dry cow therapy by assignment of four applications per DCDvet (25). They demonstrated how the nDCDvet was increased by intramammary administration with relatively little change on mg/PCU due to the low mg/kg dosage of these products.

A study on Norwegian finfish aquaculture sought to evaluate AMU by considering the total weight of AMDs prescribed divided by a DCDvet for fish (DCDvet_fish_) metric similar to the DCDvet (76). They defined the DCDvet_fish_ due to special properties related to how fish, as poikilothermic animals, consume feed at variable rates based on water temperature. As a result, the total course dose per biomass of fish was the prescribed treatment regimen rather the daily dose and number of treatment days. One DCDvet_fish_ represented the amount of a specific AMD prescribed for the treatment of 1 kg of fish under standard conditions. The nDCDVet_fish_ of an AMD was the biomass of farmed fish that could be treated with a certain amount of AMD. The annual nDCDVet_fish_ divided the total active ingredient for an AMD by the nDCDVet_fish_ for that drug. The examples of DCDvet_fish_ were 100 mg for florfenicol, 150 mg for flumequine, 800 mg for oxytetracycline, and 320 mg for trimethoprim/sulfadiazine (1:5). They used this metric to report on temporal trends for AMU of these various AMDs in the Norwegian finfish aquaculture industry, but they did not comment specifically on the use of nDCDvet_fish_ as an indicator.

The nDCDvet for finfish aquaculture is subject to the same limitations as the nUDDA and terrestrial species regarding the need for granular data and a defined ATW. Typical finfish aquaculture operations do not report the numbers of animals treated or the course length for that treatment within AMU reporting programs. The Norwegian DCDvet_fish_ presents an interesting concept, but is highly data dependent and may not be generally applicable to current AMU surveillance reporting for finfish aquaculture as these data are not available. Interestingly, the Norwegian study also stated the limitation of brood stock being excluded from their biomass estimations, similar to the issue of using total slaughter weight for the mg/PCU indicator.

### Treatment Frequency and Treatment Incidence Indicators

Treatment frequency (TF) and treatment incidence (TI) can be equated to two epidemiological measures, cumulative incidence (risk of treatment) and incidence density/intensity (rate of treatment), respectively (9). On its own, TF is the average number of treatments per animal and is simply calculated by dividing the nUDDA by the number of animals (Equation 12) (9, 44, 71, 72). The TF indicator does not give an indication of the rate of treatment, but rather how many days on average an animal in a population is treated with an active ingredient, from which the risk of treatment for an average animal can be extrapolated (9).

(12) $$\begin{array}{l} Treatment\;Frequency\;\left(doses/animal\right)\\ \;=\frac{nUDDA}{number\;of\;animals\;in\;the\;population} \end{array}$$

The TF can be used as a farm-level benchmarking indicator as it uses data from the real farm situation for actual applied dosages and true animal weights and numbers (71). Differing TFs between populations could be explained by varying disease pressures or AMU protocols if the populations are much different, making relative comparisons challenging. Germany uses TF as a benchmarking metric for AMU, calculated twice a year for all species and age groups in the country (71). One study used this approach without knowing the total quantity of active ingredients or animal weights because the nUDDA and animal numbers were available due to German law (72). The number of single applications can be extrapolated from the nUDDA indicator if the total quantity of AMDs used is known (9). The nDDDvet could also be used to calculate TF, but does not reflect the actual application of AMDs. Using the nUDDA to calculate sum of single applications would provide information on the actual number of animals treated, as well as the ability to assess each individual dose of AMD if the total amount of AMU is also recorded (72). Using nDDDvet could bias TF depending on the DDDvet and ATWs.

Treatment incidence (TI) has been defined in different ways for different purposes. Generally, it standardizes an indicator by a population time-at-risk. For example, CIPARS standardizes the nDDDvetCA by animal time-at-risk (Equation 13) (17, 26). The nDDDvetCA/1,000 animal-days-at-risk (ADR) is interesting to compare to the PCU and APCU. It presents an indicator that standardizes by both a drug dose and an actual number of animal days at risk. The denominator for Equation 13 is equivalent to the animal PCU (Equation 1) multiplied by the animal lifespan, which is the equivalent of the APCU (Equations 3 and 3.1). This presents an interesting option, but again requires that the numbers of animals, average treatment weights and lifespans are defined as accepted standards or are known from real data. The Canadian estimates from CIPARS use industry-reported ADRs (i.e., animal grow cycle lengths, or lifespans) that change annually based on data (26).

(13) $$\begin{array}{l} nDDDvetCA\;per\;1,000\;ADR\\ \;=\frac{nDDDvetCA}{total\;animals\;x\;ATW\;x\;days\;at\;risk}x\;1,000 \end{array}$$

Werner et al. (9) defined TI as the overall total amount of applied active ingredient divided by the same denominator from Equation 13, which is also equivalent to the nUDDA divided by the product of animal days at risk and the number of animals. The only difference is that the estimates from CIPARS represent the theoretical DDDvet compared to the actual AMU from the UDDA for the latter. A European study on broiler chickens calculated TI using three different methods that incorporated DDDvet, UDDAs, and the DCDvet into Equation 13 (73). The correlation between the different methods varied depending on within-flock, between-flock and between-farm comparisons, but this was not the main objective of the study. Another study on poultry farms in Vietnam found poor correlation between TI and mg AMD per reported kg biomass using mg AMD at sale or treatment (64). These results suggest that TI may reveal trends in AMU not apparent using quantity-based indicators. The discrepancies may be explained by differences in the strengths of AMD, timing of use, and variable mortality in flocks. Treatment indicence may be more balanced because it incorporates dosing and animal time-at-risk variability into its derivation, but it suffers from the same challenges inherent to the DDDvet, DCDvet and UDDA metrics.

The nDDDvetCA per 1,000 ADR is a fascinating consideration for finfish aquaculture AMU. Label dosages exist within countries for commonly used products, such as oxytetracycline and florfenicol, but neither ESVAC nor Canada have defined DDDvets for these drugs in finfish aquaculture species. The lack of defined ATWs for finfish and reported number of fish also create uncertainty for its application. Alternatively, given the link to the PCU and APCU derivations, the nDDDvetCA for finfish could use the APCU denominator to derive this estimate of TI for finfish if the total slaughter weight and lifespan are used to estimate the APCU. Given that farm-level data are typically not available, the alternative TI estimates based on UDDA or DCDA are not available for finfish aquaculture.

## Discussion—An Example Application of AMU Indicators to Finfish Aquaculture Data

Tables 2, 3 demonstrate the application of the different AMU indicators to a hypothetical finfish population and florfenicol AMU data. In Canada, the label dose for florfenicol in fish is 10 mg/kg/day with a treatment course of 10 days (77). This example illustrates how the calculations are performed and highlights the data limitations for their application when considering the availability of international, and particularly Canadian, finfish data. It is common practice for countries to report annual slaughter weights and total kg of AMD used in their finfish industries. Conversely, they do not collect data on or report the numbers of fish or drug dosages by individual fish. These are not easy pieces of information to glean from typical production records as fish are grown and managed in pens. The concept of ATW is also confusing as its hypothetical meaning is often at odds with typical industry practice for reasons already discussed (43). As a result, national finfish AMU estimates are often standardized by a PCU for biomass that is based on the total annual slaughter weight of fish (49–51, 55).

**Table 2:** Hypothetical data for derivation of antimicrobial use indicators for finfish aquaculture.

**Variable**	**Value**	**Considerations**
Number of fish	100,000	Unknown—countries typically do not report
Average treatment weight (kg)^*^	2.5	Value not described for Canadian or global industry
Total fish slaughter weight (kg)	500,000	Countries commonly report
Marine grow-out cycle length (years)	1.5	Estimate for Canadian west coast Atlantic salmon
Antimicrobial active ingredient	Florfenicol	Labeled for use in Canadian finfish
DDDvetCA_florfenicol_ (mg/kg/day)	10	Canadian label dose for florfenicol
Dose course for florfenicol (days)	10	Canadian label dose for florfenicol
Number of fish treated	50,000	Unknown—countries do not report
Average actual weight at treatment (kg)	1.0	Unknown and variable with region, disease
Total florfenicol used (kg)	5,000	10 d ^*^ 10 mg/kg ^*^ 50,000 fish ^*^ 1 kg

* *Average treatment weight (kg) = hypothetical average of the slaughter (5 kg) and starting weights (0 kg) per Montforts and Tarazona Lafarga (41)*.

*DDDvetCA, Defined Daily Dose Animal for Canadian animals*.

**Table 3:** Application of antimicrobial use indicators to finfish aquaculture data.

**Metric/Indicator**	**Value**	**Considerations**
mg/PCU_average treatment weight_ (mg drug/kg biomass)	20	=5,000 kg/(100,000 fish ^*^ 2.5 kg)
mg/PCU_total slaughter weight_ (mg drug/kg biomass)	10	=5,000 kg/500,000 kg
mg/APCU_average treatment weight_ (mg drug/kg biomass)	13.33	=5,000 kg/(100,000 fish ^*^ 2.5 kg ^*^ 1.5 years)
mg/APCU_total slaughter weight_ (mg drug/kg biomass)	6.67	=5,000 kg/(500,000 kg ^*^ 1.5 years to market kg)^*^
nDDDvetCA_florfenicol_	200,000	= 5,000 kg/(10 mg/kg/day ^*^ 2.5 kg)
DCDvet_florfenicol_ (mg/kg)	100	= DDDvet_florfenicol_ 10 mg/kg ^*^ course 10 d
nDCDvet_florfenicol_ (# of treatment courses)	20,000	= 5,000 kg/(DCDvet_florfenicol_ 100 mg/kg ^*^ 2.5 kg)
UDDA_florfenicol_ (mg/fish/day)	10	=5,000 kg/(50,000 fish ^*^ 10 days)
UDDA_kg florfenicol_ (mg/kg of fish/day)	10	=5,000 kg/(50,000 fish ^*^ 1.0 kg ^*^ 10 days)
nUDDA_florfenicol_ (# used daily doses)	500,000	=50,000 fish ^*^ 1 drug ^*^ 10 days
Treatment frequency_total population_ (doses/fish)	5	=nUDDA/100,000 fish
Treatment frequency_treated population_ (doses/fish)	10	=nUDDA/50,000 fish
Treatment incidence (nDDDvetCA per 1,000 ADR)	0.73	=nDDDvetCA_florfenicol_/(500,000 kg ^*^ 1.5 years ^*^ 365/1,000 days)^**^

* *Total grow-out length mg/APCU_total slaughter weight_–the slaughter weight was multiplied by the average grow-out cycle length to give the life-adjusted slaughter weight*.

** *The nDDDvetCA per 1,000 animal days at risk used the APCU for total slaughter weight as the denominator and scaled per 1,000 fish days at risk*.

*PCU, population correction unit; APCU, adjusted PCU; nDDDvetCA, number of Defined Daily Doses animal for Canadian data; UDDA, Used Daily Doses Animal; UDDA_kg_, UDDA/kg of animal; ADR, animal days at risk*.

These hypothetical data illustrate the differences when using total slaughter weight compared to an ATW and number of fish for the mg/PCU and mg/APCU indicators. Based on this example, the use of total slaughter weight reduces the resulting indicator for both by approximately half. The length of the grow-out cycle also decreases the mg/APCU indicator compared to the mg/PCU. If the objective for estimating AMU is to compare between countries, it is best to use common and consistent PCU for biomass. The availability of total slaughter weight is a transparent and consistent approach for finfish AMU comparison using the mg/PCU indicator. However, it precludes direct comparison of mg/PCU or mg/APCU to terrestrial animals within a country such as Canada where the latter is based on animal numbers and average treatment weights (26). The lack of a defined ATW for finfish creates problems for the derivation of the nDDDvet, nDCDvet and nDDDvetCA per 1,000 ADR indicators. The lack of ESVAC or internationally recognized DDDvet values for common finfish aquaculture antimicrobials also presents challenges for standardized nDDDvet estimates (65, 66). As is the case in Canada, it is common for countries to define their own values, such as the nDDDvetCA that reflect their region-specific drug labels (26). The lack of granular, farm-level data precludes the ability to estimate the nDCDvet. The link between the formula for nDDDvetCA per 1,000 ADR and the APCU does allow for the alternative calculation of this estimate of TI for finfish, whereby the APCU based on either total slaughter weight or ATW, and marine grow-out lifespan is used for calculation. This does allow for an estimate of TI for finfish, but again suffers from the lack of an international DDDvet for florfenicol and the need to estimate an ATW.

Another important consideration is that a PCU based on the total annual slaughter weight for finfish does not account for existing, live fish in the population at a given time or the fish that die. It excludes brood stock and any freshwater grow-out phase for a given fish species. This is similar to the PCU approach to poultry whereby breeder stock are not included in the calculations as is time spent in the hatchery. Generally, the mg/PCU indicator does not account for drug potency, but species-level comparisons are possible if the data are available (21, 25, 63). The DDDvet accounts for varying drug doses/indications and AMU at more granular levels such as animal species and breed (34), but is limited in application for finfish without DDDvet and ATWs. Unfortunately, the DCDvet metric is highly data dependent with high resource demands such as dose and indication information for AMU and species or animal standard weights (34, 44). These are not available in finfish aquaculture at this time. While the DDDvet has become a popular standard metric in the EU, the PCU is still used by over 25 countries as a means of AMU standardization (34). With this in mind, the PCU is likely to hold high importance for finfish aquaculture at this time. The APCU shows some utility, but requires further investigation and consideration of its derivation from reports of total annual slaughter mass for finfish and weight of antimicrobials used.

This review adds important information about the application of AMU metrics and indicators to finfish aquaculture AMU and production. Werner et al. (9) reviewed AMU metrics and indicators broadly for all animals, with a focus on terrestrial animals. There was no discussion about specific application to and data sources for global finfish aquaculture AMU and production data. This review provides this specific finfish aquaculture lens as this industry continues to grow in importance as a global protein source. In particular, the papers included in the review discuss the PCU metric and mg/PCU indicator, which are increasingly used as reporting tools for national surveillance programs such as CIPARS and ESVAC (26, 55) and recent global aquaculture AMU projections (57) based on FAO production statistics (54). Radke (34) proposed the APCU metric as a means to consider the variable lengths of life of different food animal species. New data from a census of AMU for 2.6 million feedlot cattle in western Canada also explored the nDDDvet, mg/100 cattle-at-risk, and mg/PCU indicators, with resulting differences in results between them (21, 31). This review also explores the potential difficulties and confusions when defining and applying ATW values for food animal species. This review will support future work to consider the application of these indicators and metrics to finfish aquaculture AMU data as the pressure increases on this industry to demonstrate antimicrobial stewardship.

## Conclusions

There is no single AMU indicator that is suitable for all intended purposes for surveillance and reporting of finfish aquaculture AMU data. This review highlights the common AMU indicators and inherent AMU and population metrics developed for different purposes in terrestrial animal livestock production and surveillance. Specific consideration for the finfish aquaculture industry illustrates that the mg/PCU based on total annual slaughter weight is common, consistent and transparent for international comparisons for trade or stewardship assessment purposes. Further work on ATWs and DDDvets is required to be able to apply other indicators to the industry with confidence. This work is required for further assessment of antimicrobial stewardship, farm-to-farm comparison, or should it become required, benchmarking. Ultimately, the indicator used should be fit-for-purpose in that it must satisfy the objective of the surveillance program and motivation for comparison. It must also provide meaningful and useful data back to the industry and other stakeholders. Commonly available data for finfish aquaculture, such as total slaughter weight, present an alternative biomass estimate for the industry to calculate mg/PCU for AMU. This strategy may miss some fish biomass in the system, but provides for a common denominator that can be applied across the major finfish producing countries that report these and AMU data. This allows for international comparisons of mg/PCU indicators of AMU, whether it be to inform antimicrobial stewardship policy or trade decisions. The PCU concept of ATW continues to present challenges for industry interpretation as it often does not reflect actual industry AMU practice. We argue that the term “average biomass” is a better reflection of what the value actually represents. Ultimately, the mg/PCU and mg/APCU indicators provide the means for international comparison of finfish aquaculture AMU. The ability to use nDDDvet or nDDDvet per animal-days-at-risk will be limited until progress is made to define an ATW. This will require industry engagement and buy in, which is crucial if AMU reporting and estimation is to be deemed credible and provide value back to the finfish aquaculture industry.

## Author Contributions

JN development and execution of the search strategy (90%), article screening (100%), data extraction (50%), writing a detailed, draft report on AMU surveillance metrics and indicators submitted to the British Columbia Ministry of Agriculture in March 2020 (75%), and review and writing of the manuscript (15%). SO conception of the work (50%), guided development of the review search strategy (10%), writing of the report to British Columbia (15%), and primary lead for writing and revising the manuscript (60%). BR conception of the work (50%), review and writing of the report (10%), and review and writing of the manuscript (10%). DP and PH review and writing of the manuscript (5%). AB data extraction (50%), reference management, review, and writing of the manuscript (5%). All authors contributed to the article and approved the submitted version.

## Conflict of Interest

The authors declare that the research was conducted in the absence of any commercial or financial relationships that could be construed as a potential conflict of interest.
