# Supplementary material for: Antimicrobial Use Surveillance Indicators for Finfish Aquaculture Production: A Review
Source: Front Vet Sci. 2021 Mar 11;8:595152. doi: 10.3389/fvets.2021.595152 (PMC7991786; doi:10.3389/fvets.2021.595152)
Supplement: Supplementary file 1 [file Data_Sheet_1.pdf]

frontiers

# *Supplementary Material*

## 1 Literature Review Strategy

### 1.1 Timeline and scientific databases

The search was executed on January 20, 2020 and included literature from January 1, 2016 to present to update a recent review on AMU for general animal surveillance published by Werner, et al., 2018. Communication with the authors indicated that their search was completed in early 2015. The search included five scientific databases:

1. Medline® via Ovid® provides literature (from 1946 – present). It is the world's leading bibliographic source for biomedical scholarly literature and research.
2. CAB Abstracts® via Web of Science™ covers multi-disciplinary literature (from 1910 - present) in the fields of nature, health, and social sciences. CAB Abstracts is the leading English-language bibliographic information service providing access to the world's applied life sciences literature.
3. Embase via Ovid® covers a vast range of biomedical sciences journals, with an added focus on European studies (from 1974 – present) not present in MEDLINE®.
4. AGRICOLA ™ via ProQuest® sources from the United States National Agricultural Library to retrieve global literature (from 1970 – present) on the topic of agriculture.
5. BIOSIS Previews via Web of Science™ covers pre-clinical and experimental research, methods and instrumentation, animal studies, and more (1926 - present).

### 1.2 Search strategies and results

Medline® via Ovid®

| Component | Search Terms | # Results |
|---|---|---|
| 1. Surveillance | (surveillance* or inspect* or control* or metric* or measure* or observ* or scrutin* or examin*or monitor* or track* or evaluat*).ti,ab,kw. [mp=title, abstract, original title, name of substance word, subject heading word, floating sub-heading word, keyword heading word, organism supplementary concept word, protocol supplementary concept word, rare disease supplementary concept word, unique identifier, synonyms] | 10320274 |
| 2. Use | ("Use*" or Usage* or Treat* or Appli* or Prescribe* or admin* or distribut* or sell* or sale* or metric or metrics or distribut*).ti,ab,kw. [mp=title, abstract, original title, name of substance word, subject heading word, floating sub-heading word, keyword heading word, organism supplementary concept word, protocol supplementary | 12242908 |

| | | |
|---|---|---|
| | concept word, rare disease supplementary concept word, unique identifier, synonyms] | |
| 3. Antimicrobial | (antibiotic* or antimicrobial* or anti-microbial* or anti-biotic* or anti-bacterial* or antibacterial* OR multidrug or medication* or drug* or antiinfective or anti-infective or anti-infective agent*).ti,ab,kw. [mp=title, abstract, original title, name of substance word, subject heading word, floating sub-heading word, keyword heading word, organism supplementary concept word, protocol supplementary concept word, rare disease supplementary concept word, unique identifier, synonyms] | 2230687 |
| 4. Metric | (PDD or prescribed daily dose or ACD or animal course dose or DCD or defined course dose or DCDA or defined course dose animal or ADDD or animal defined daily dose or DDDA or defined daily dose animal or DDD or UDD or UCD or PCU or daily defined dose or daily course dose or population correct* unit or used daily dose or APCU or adjusted population correct* unit or DOT or DPD or Daily Product Dose or treatment incidence or Treatment frequency or treatment incidence rate or treatment frequency or sale* data or product related daily dose or animal daily dose).ti,ab,kw. | 55775 |
| 5. Animal | (Cattle or cow or bull or bulls or steer or calf or calves or bos taurus or beef or veal or pig or piglet or swine or hog or sow or pork or sus scrofa domesticus or chick or chicken or chickens or rooster or hen or broiler or gallus gallus domesticus or turkeys or meleagris gallopavo or turkey or gobbler or poultr*) or ((farm* or domestic or aquaculture or livestock) and (fish* or finfish or fin-fish or atlantic salmon or pacific salmon or arctic char or black cod or chinook salmon or coho or tilapia)).ti,ab,kw. | 956412 |
| 6. 1 AND 2 AND 3 AND 4 AND 5 | | 129 |

| | | |
|---|---|---|
| 7. limit 6 to yr="2016 - Current" | | 60 |

Embase® via Ovid®

| Component | Search Terms | # Results |
|---|---|---|
| 1. Surveillance | (surveillance* or inspect* or control* or metric* or measure* or observ* or scrutin* or examin*or monitor* or track* or evaluat*).ti,ab,kw. [mp=title, abstract, original title, name of substance word, subject heading word, floating sub-heading word, keyword heading word, organism supplementary concept word, protocol supplementary concept word, rare disease supplementary concept word, unique identifier, synonyms] | 13233562 |
| 2. Use | ("Use*" or Usage* or Treat* or Appli* or Prescribe* or admin* or distribut* or sell* or sale* or metric or metrics or distribut*).ti,ab,kw. [mp=title, abstract, original title, name of substance word, subject heading word, floating sub-heading word, keyword heading word, organism supplementary concept word, protocol supplementary concept word, rare disease supplementary concept word, unique identifier, synonyms] | 15503048 |
| 3. Antimicrobial | (antibiotic* or antimicrobial* or anti-microbial* or anti-biotic* or anti-bacterial* or antibacterial* or multidrug or medication* or drug* or antiinfective or anti-infective or anti-infective agent*).ti,ab,kw. [mp=title, abstract, original title, name of substance word, subject heading word, floating sub-heading word, keyword heading word, organism supplementary concept word, protocol supplementary concept word, rare disease supplementary concept word, unique identifier, synonyms] | 3101711 |
| 4. Metric | (PDD or prescribed daily dose or ACD or animal course dose or DCD or defined course dose or DCDA or defined course dose animal or ADDD or animal defined daily dose or DDDA or defined daily dose animal or DDD or UDD or UCD or PCU or daily defined dose or daily course dose or population correct* unit or used daily dose or APCU or adjusted population correct* unit or DOT or DPD or Daily | 72770 |

| | | |
|---|---|---|
| | Product Dose or treatment incidence or Treatment frequency or treatment incidence rate or treatment frequency or sale* data or product related daily dose or animal daily dose).ti,ab,kw. | |
| 5. Animal | (Cattle or cow or bull or bulls or steer or calf or calves or bos taurus or beef or veal or pig or piglet or swine or hog or sow or pork or sus scrofa domesticus or chick or chicken or chickens or rooster or hen or broiler or gallus gallus domesticus or turkeys or meleagris gallopavo or turkey or gobbler or poultr*) or ((farm* or domestic or aquaculture or livestock) and (fish* or finfish or fin-fish or atlantic salmon or pacific salmon or arctic char or black cod or chinook salmon or coho or tilapia)).ti,ab,kw. | 897833 |
| 6. 1 AND 2 AND 3 AND 4 AND 5 | | 148 |
| 7. limit 6 to yr="2016 - Current | | 62 |

AGRICOLA ™ via ProQuest

| Component | Search Terms | # Results |
|---|---|---|
| 1. Surveillance | noft(surveillance* or inspect* or control* or metric* or measure* or observ* or scrutin* or examin*or monitor* or track* or evaluat*) | 1555184 |
| 2. Use | noft("Use*" or Usage* or Treat* or Appli* or Prescribe* or admin* or distribut* or sell* or sale* or metric or metrics or distribut*) | 2184459 |
| 3. Antimicrobial | noft(antibiotic* or antimicrobial* or anti-microbial* or anti-biotic* or anti-bacterial* or antibacterial* or multidrug or | 262309 |

| | | |
|---|---|---|
| | medication* or drug* or antiinfective or anti-infective or anti-infective agent*) | |
| 4. Metric | noft(PDD or prescribed daily dose or ACD or animal course dose or DCD or defined course dose or DCDA or defined course dose animal or ADDD or animal defined daily dose or DDDA or defined daily dose animal or DDD or UDD or UCD or PCU or daily defined dose or daily course dose or population correct* unit or used daily dose or APCU or adjusted population correct* unit or DOT or DPD or Daily Product Dose or treatment incidence or Treatment frequency or treatment incidence rate or treatment frequency or sale* data or product related daily dose or animal daily dose) | 47031 |
| 5. Animal | noft(Cattle or cow or bull or bulls or steer or calf or calves or bos taurus or beef or veal or pig or piglet or swine or hog or sow or pork or sus scrofa domesticus or chick or chicken or chickens or rooster or hen or broiler or gallus gallus domesticus or turkeys or meleagris gallopavo or turkey or gobbler or poultr*) or ((farm* or domestic or aquaculture or livestock) and (fish* or finfish or fin-fish or atlantic salmon or pacific salmon or arctic char or black cod or chinook salmon or coho or tilapia)) | 668750 |
| 6. 1 AND 2 AND 3 AND 4 AND 5 | | 914 |
| 7. Filter 2016-01-01 – 2020-01-01 | | 198 |

CAB Abstracts® via Web of Science™

| Component | Search Terms | # Results |
|---|---|---|

| | | |
|---|---|---|
| 1. Surveillance | Topic: ((surveillance* or inspect* or control* or metric* or measure* or observ* or scrutin* or examin*or monitor* or track* or evaluat*)) | 4962453 |
| 2. Use | Topic: (("Use*" or Usage* or Treat* or Appli* or Prescribe* or admin* or distribut* or sell* or sale* or metric or metrics or distribut*)) | 5715468 |
| 3. Antimicrobial | Topic: ((antibiotic* or antimicrobial* or anti-microbial* or anti-biotic* or anti-bacterial* or antibacterial* or multidrug or medication* or drug* or antiinfective or anti-infective or anti-infective agent*)) | 1587251 |
| 4. Metric | Topic: ((PDD or prescribed daily dose or ACD or animal course dose or DCD or defined course dose or DCDA or defined course dose animal or ADDD or animal defined daily dose or DDDA or defined daily dose animal or DDD or UDD or UCD or PCU or daily defined dose or daily course dose or population correct* unit or used daily dose or APCU or adjusted population correct* unit or DOT or DPD or Daily Product Dose or treatment incidence or Treatment frequency or treatment incidence rate or treatment frequency or sale* data or product related daily dose or animal daily dose)) | 163423 |
| 5. Animal | Topic: ((Cattle or cow or bull or bulls or steer or calf or calves or bos taurus or beef or veal or pig or piglet or swine or hog or sow or pork or sus scrofa domesticus or chick or chicken or chickens or rooster or hen or broiler or gallus gallus domesticus or turkeys or meleagris gallopavo or turkey or gobbler or poultr*) or ((farm* or domestic or aquaculture or livestock) and (fish* or finfish or fin-fish or atlantic salmon or pacific salmon or arctic char or black cod or chinook salmon or coho or tilapia))) | 1774047 |
| 6. 1 AND 2 AND 3 AND 4 AND 5 | | 7658 |

| | | |
|---|---|---|
| 7. 6 limited to 2016-2020 | | 1055 |

Biosis® via Web of Science™

| Component | Search Terms | # Results |
|---|---|---|
| 1. Surveillance | Topic: ((surveillance* or inspect* or control* or metric* or measure* or observ* or scrutin* or examin*or monitor* or track* or evaluat*)) | 10101482 |
| 2. Use | Topic: (("Use*" or Usage* or Treat* or Appli* or Prescribe* or admin* or distribut* or sell* or sale* or metric or metrics or distribut*)) | 12241802 |
| 3. Antimicrobial | Topic: ((antibiotic* or antimicrobial* or anti-microbial* or anti-biotic* or anti-bacterial* or antibacterial* or multidrug or medication* or drug* or antiinfective or anti-infective or anti-infective agent*)) | 5126564 |
| 4. Metric | Topic: ((PDD or prescribed daily dose or ACD or animal course dose or DCD or defined course dose or DCDA or defined course dose animal or ADDD or animal defined daily dose or DDDA or defined daily dose animal or DDD or UDD or UCD or PCU or daily defined dose or daily course dose or population correct* unit or used daily dose or APCU or adjusted population correct* unit or DOT or DPD or Daily Product Dose or treatment incidence or Treatment frequency or treatment incidence rate or treatment frequency or sale* data or product related daily dose or animal daily dose)) | 457235 |
| 5. Animal | Topic: ((Cattle or cow or bull or bulls or steer or calf or calves or bos taurus or beef or veal or pig or piglet or swine or hog or sow or pork or sus scrofa domesticus or chick or chicken or chickens or rooster or hen or broiler or gallus gallus domesticus or turkeys or meleagris gallopavo or turkey or gobbler or poultr*) or ((farm* or domestic or aquaculture or livestock) and (fish* or finfish or fin-fish or atlantic salmon or pacific salmon or arctic char or black cod or chinook salmon or coho or tilapia)) | 1391050 |

| | | |
|---|---|---|
| | | |
| 6. 1 AND 2 AND 3 AND 4 AND 5 | | 3909 |
| 7. 6 limited to 2016-2020 | | 562 |

# 2 Supplementary Figures and Tables

## 2.1 Supplementary Figures

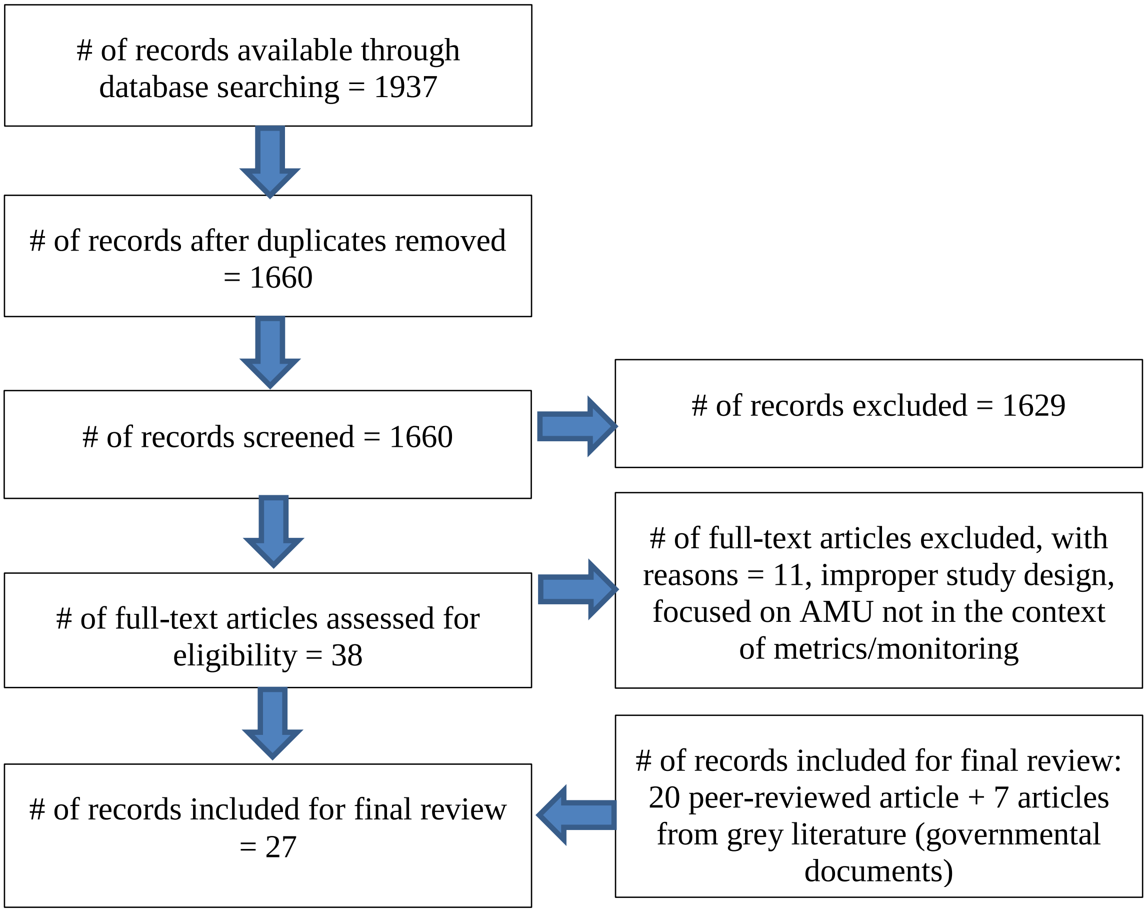


**Supplementary Figure 1.** The compiled results from the search of five scientific databases and internet search engines, and screening of articles that included information about antimicrobial use surveillance metrics and indicators in animals (January 1, 2016 to January 20, 2020).

## 2.2 Supplementary Tables

**Supplementary Table 1. Average treatment weights used to calculate Population Correction Units for various terrestrial animal species (adapted from the European Medicines Agency (2019) and Radke (2017).**

| Animal Category | Average treatment weights | |
|---|---|---|
| | ESVAC weights (kg)[a,b] | Adjusted weights (kg)[b] |
| **Pigs** | | |
| Suckling piglets | 4 | 4 |
| Weaner pigs | 12 | 12 |
| Sows/boars | 240 | 240 |
| Slaughter pigs | 65 (25-105)* | 65 |
| Finisher | 65 | 65 |
| Imported/exported pigs for slaughter | 65 | 65 |
| Imported/exported pigs for fattening | 25 | - |
| **Cattle** | | |
| Slaughter cows | 500 | 627 |
| Slaughter heifers | 200 | 269 (45-493)* |
| Slaughter bullocks and bulls | 500 | 329 (45-612)* |
| Slaughter calves and young cattle | 140 | 169 (45-293)* |
| Imported/exported cattle for slaughter | 500 | 299 |
| Imported/exported cattle for fattening | 140 | 169 (45-293)* |
| Livestock dairy cows | 500 | 627 |
| Veal calves | 80 | 80 |
| **Poultry** | | |
| Slaughter broilers | 1 | 1 |
| Slaughter turkeys | 6.5 | 6.5 |
| Imported/exported broilers for slaughter | 1 | 1 |
| **Finfish** | | |
| Slaughter fish | ***Total slaughter weight (kg)*** | |

[a] (European Medicines Agency, 2019)

[b] (Radke, 2017)

* Weight used (weight range for category in brackets), where applicable.

**Supplementary Table 2. Complete list of articles and their extracted data that were included in the final review.**

See separate excel file titled “2021-01-21-SupplTable2-reviewedarticles-dataextraction.xlsx”.
